# Moisture Behavior of Pharmaceutical Powder during the Tableting Process

**DOI:** 10.3390/pharmaceutics15061652

**Published:** 2023-06-04

**Authors:** Komlan Koumbogle, Ryan Gosselin, François Gitzhofer, Nicolas Abatzoglou

**Affiliations:** Department of Chemical and Biotechnological Engineering, Université de Sherbrooke, 2500 Boulevard de l’Université, Sherbrooke, QC J1K 2R1, Canada; komlan.koumbogle@usherbrooke.ca (K.K.); ryan.gosselin@usherbrooke.ca (R.G.)

**Keywords:** tableting, COMSOL Multiphysics, finite element analysis, compaction, sticking, moisture, Pharmaceutics, Drucker–Prager cap (DPC), heat, air and moisture (HAM), temperature field, relative density, NIR penetration depth

## Abstract

The moisture content of pharmaceutical powder is a key parameter contributing to tablet sticking during the tableting process. This study investigates powder moisture behavior during the compaction phase of the tableting process. Finite element analysis software COMSOL Multiphysics^®^ 5.6 was used to simulate the compaction microcrystalline cellulose (VIVAPUR PH101) powder and predict temperature and moisture content distributions, as well as their evolution over time, during a single compaction. To validate the simulation, a near-infrared sensor and a thermal infrared camera were used to measure tablet surface temperature and surface moisture, respectively, just after ejection. The partial least squares regression (PLS) method was used to predict the surface moisture content of the ejected tablet. Thermal infrared camera images of the ejected tablet showed powder bed temperature increasing during compaction and a gradual rise in tablet temperature along with tableting runs. Simulation results showed that moisture evaporate from the compacted powder bed to the surrounding environment. The predicted surface moisture content of ejected tablets after compaction was higher compared to that of loose powder and decreased gradually as tableting runs increased. These observations suggest that the moisture evaporating from the powder bed accumulates at the interface between the punch and tablet surface. Evaporated water molecules can be physiosorbed on the punch surface and cause a capillary condensation locally at the punch and tablet interface during dwell time. Locally formed capillary bridge may induce a capillary force between tablet surface particles and the punch surface and cause the sticking.

## 1. Introduction

Tableting is the mechanical process adopted by the pharmaceutical industry to produce medicinal tablets. It involves direct compression of a powder mixture in a die using three steps: die filling, during which the formulation is delivered to the die cavity; compaction, during which pressure is applied to the formulation; and ejection, when the compacted tablet is ejected from the die cavity [1]. Up to 1 million tablets can be pressed per hour with one multi-die rotating press. The formulation compacted during the process is a mixture of active principal ingredients and excipients, such as ligands, binders, and lubricants, with a given moisture content.

The study of the physics of the tableting process is of great interest in order to understand the mechanism of consolidation of the granules during compaction, the punch-sticking phenomena, and the quality management of the pressed product. Several mathematical equations, such as Hekel’s [2], Kawakita’s [3], and Leuenberger’s [4] equations, have been developed to investigate the ability of formulations to flow, deform, and consolidate under pressure. These equations are usually used to predict how a formulation performs during the tableting process and minimize defects related to the pressing process. Although these equations are useful for preparing the best formulations for the tableting process, they do not provide a detailed insight of the phenomena occurring in the whole volume of the powder bed during the compaction process. Hence, simulation models based on the finite element method (FEM) have been developed to analyze in greater detail the powder bed behavior during compaction [5,6,7,8]. These phenomenological constituent methods are based on experimental calibrations using the variable physical properties of the compressed material. Several constitutive models have been developed, of which the DiMaggio–Sandler model [9], the Cam-Clay model [10], and the Drucker–Prager Cap (DPC) model [5,6] are well known. The DPC model is the most used for the pharmaceutical tableting process due to its ability to describe physical phenomena taking place during compression of a granular medium. It was first developed to investigate irreversible deformations of soils during compaction [5]. The DPC model was subsequently modified and adapted to pharmaceutical powder, and a calibration method was proposed to define all the involved parameters [11,12]. This modified DPC model was then used to study the mechanical behavior of powders, such as strain, stress, and density distribution, in the compact during compaction [13,14,15,16,17]. Thermomechanical analysis using the modified DPC model described how the heat generated during compaction progressively increased the powder bed temperature in all its volume [18,19,20] and was conclusive, along with results from previous experimental work [21,22,23].

During the tableting process, defects such as cracking, stretching, capping, lamination, chipping, restricting, and sticking may be present in the finally ejected tablets [1]. Coupling the FEM with the modified DPC allows determination of how the defects developed during compaction and helps to improve the understanding of the tableting process accordingly. Defects such as capping, cracking, lamination, and chipping have been intensively investigated and have been shown to originate from the inhomogeneous distribution of stress, relative density, and buildup of air pressure in the powder bed during compaction [24,25,26,27]. Furthermore, defects such as sticking, which is a persistent problem during the tableting process, were investigated to discover how the tableting process parameters, compressed material, compression tool properties, and ambient conditions contributed to the sticking defects [28,29,30,31,32,33,34,35,36]. Multivariate analysis, adhesion force measurement, push-off force measurements, material accumulation quantification, and the discrete element method have often been used for testing the propensity to stick [37,38,39,40,41,42,43]. (1) These studies identified the van der Waals forces and capillary forces to play a dominating role in the observed adhesion. The interplay between the interparticle cohesive force and wall–powder adhesion force results in localized attachment of the surface granules or layer of the tablet to the surface of the compression tool. Tableting force and speed, moisture level of the formulation, and properties of the compressed material govern the cohesivity of the tablet, and hence the tendency to stick. (2) From a phenomenological point of view, an increase in granule temperature during compaction may lead to the melting of some ingredients, such as ibuprofen and magnesium stearate, or of eutectic components present in the formulation, which may contribute to sticking. However, other studies have invalidated the melting hypothesis by showing that sticking still occurs when high-melting-point material, such as ibuprofen sodium, are compressed [44]. (3) Recent multivariate analysis of granulation and tableting process parameters [41,43] showed that the moisture level of the formulation plays a key role in the occurrence of sticking. While the results obtained from these studies provide good data to understand sticking, it is of interest to identify the phenomena that can trigger the development of sticking during compaction.

Near-infrared (NIR) spectroscopy is often used in the pharmaceutical industry as a faster, cheaper, and nondestructive way of analyzing pharmaceutical products. Its use is encouraged in process analytical technology (PAT) for on- and inline measurements and control of pharmaceutical products. The overall objective of its use is to probe a sample to acquire qualitative and/or quantitative information about the interaction between NIR electromagnetic waves and the sample’s constituents. NIR spectroscopy has been employed to investigate H-bonds and hydration of various molecules, such as alcohols, fatty acids, amides, polymers, and water [45,46]. In the NIR region, (1) bands arising from overtones and combinations of O-H, N-H and C-H vibrations appear strongly due to their larger anharmonicity, and (2) large band shifts are induced by the formation of hydrogen bonds and hydration. Within the NIR wavelength range, i.e., 700 to 2500 nm, prominent absorption bands of liquid water are found at 760, 970, 1190, 1450, and 1940 nm [47,48]. These absorption bands are due to the second overtone of the OH stretching band (3ν1,3), the combination of the first overtone of the O-H stretching band and OH-bending band (2ν1,3 + ν2), the first overtone of the OH-stretching band (2ν1,3), and the combination of the OH-stretching band and O-H bending band (2ν1,3 + ν2) [49]. Hence, NIR spectroscopy has been used to measure the moisture content of pharmaceutical tablets and powders during granulation, drying, and the tableting process [46,50,51]. The quantification of moisture content is often based on an experimental calibration model obtained with partial least squares (PLS) regression, multiple linear regression, or principal component regression. The calibration model thus established is then used to predict the inline or offline moisture content of powder or tablets during such pharmaceutical processes as wet granulation or tableting [52,53,54].

This study aimed to investigate phenomenologically the buildup of the sticking phenomenon during the compaction of pharmaceutical powder through the simulation of powder moisture behavior under compression. The AFEM method, based on the modified DPC model, was used to investigate moisture transport and thermomechanical behavior of powder during the tableting process, particularly during the compaction phase. The evolution and distribution of powder bed density, temperature, and moisture changes during the compaction process were studied. We hypothesized that the migration of powder moisture toward compression tools due to temperature and local humidity gradients contributes to the development of sticking. The results of this study show how thermomechanical phenomena can lead to moisture migration and potentially further induce sticking through capillary forces. To validate the simulation results, the temperature of the tablet surface and sides and the surface moisture content of the tablet a second after ejection were measured using PAT tools, such as a thermal infrared camera and NIR sensor. The results of this study provide new and original insights into the phenomenology of the sticking defect observed during the tableting process.

## 2. Materials and Methods

### 2.1. Materials

Microcrystalline cellulose (MCC) grade VIVAPUR PH101 (JRS Pharma, LP., Patterson, New York, NY, USA) was used as the model powder material. The bulk density of the loose powder was measured with a 100 mL beaker and was found to be 279 ± 2.5 kg/m^3^. The true density of the dry, solid particles of MCC ranges between 1512 and 1668 kg/m^3^ [55], and hence a value of 1570 kg/m^3^ [20] was adopted in this study. The powder was stored at a controlled humidity level of 33.5 ± 0.1% and a temperature of 21.1 ± 0.5 °C. The water content of the powder was measured with an analytical transmitter (Mettler M100, Mettler-Toledo Ltd., Leicester, UK), which dries the powder with an infrared source and calculates the water content by mass difference. The mean of three measurements was calculated, and the value obtained for water content was 4.5 ± 0.3%. Water content density of the powder in the die before compaction was obtained by the following formula:

(1) $$\rho_{app\_w}=\rho_{app}*w$$

where $\rho_{app\_w}$ is the apparent moisture content density (kg/m^3^), $\rho_{app}$ is the apparent powder density in the die before compaction and w is the moisture content (%) in the powder before compaction. With a powder mass of 500 mg at the maximum capacity of the compression die (height = 12 mm, diameter = 11 mm), the apparent density of the powder before compaction was 438.6 kg/m^3^. The mean value of apparent moisture density obtained from the three measurements of moisture content was 20.85 ± 1.2% kg/m^3^.

### 2.2. Methods

#### 2.2.1. Heat Capacity of the Material

The heat capacity (Cp) of the powder was determined with a differential scanning calorimetry apparatus NETZSCH DSC 404F3 (NETZSCH-Gerätebau GmbH, Wittelsbacherstraße 42, Selb, Germany) with platinum as the reference material. The test powder sample weighed 23.7 mg, and the measurements were taken under a N_2_ atmosphere with the temperature ranging from 29 °C to 130 °C and a heating rate of 5 °C/min. The heat capacity in this study was constant during powder compression.

#### 2.2.2. Thermal Diffusivity and Conductivity

**Figure 1:**
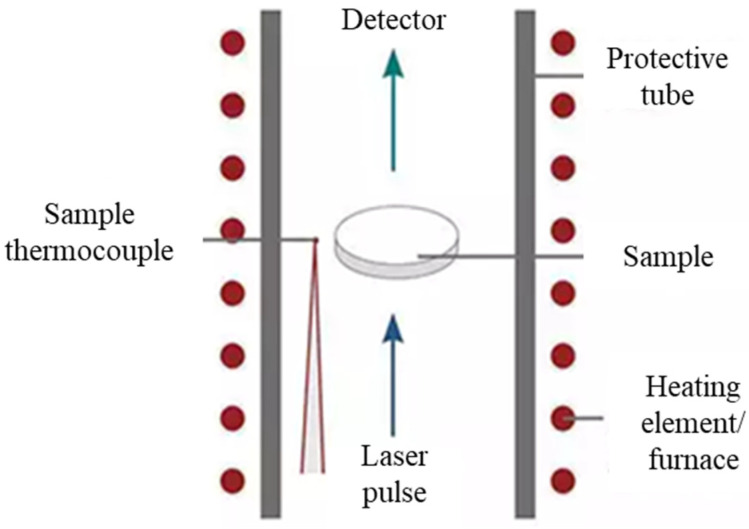
Diagram describing the laser flash principle.

The thermal diffusivity of MCC PH101 powder compacted at 200 MPa pressure with a 12-ton manual hydraulic press (Carver) was measured with a laser flash analysis apparatus NETZSCH LFA 457 (NETZSCH-Gerätebau GmbH, Wittelsbacherstraße 42, Selb, Germany). The test sample thickness was 1.71 mm and the diameter was 25 mm. Measurements were taken under a N_2_ atmosphere at a temperature ranging from 29 °C to 130 °C and a heating rate of 5 °C/min. A neodymium glass laser with a wavelength of 1054 nm was used with an energy of 10 J per pulse each 0.3 ms and a voltage of 2978 V. The measurement principle is illustrated on Figure 1. The front surface of the plane-parallel sample was heated by a laser pulse, and the resulting temperature increase at the sample’s rear face was recorded as a function of time. In a one-dimensional heat flow, thermal diffusivity was calculated with this temperature rise as follows [56]:

(2) $$\alpha =0.1338\frac{d^{2}}{t_{0.5}}$$

where *α* was thermal diffusivity in m^2^/s, *d* was sample thickness in m, and *t*_0.5_ was the half-time (time value at half-signal height) in seconds.

From the measured thermal diffusivity and heat capacity, thermal conductivity (k) was calculated with the following equation:

(3) $$\lambda ={\alpha \rho_{app}C}_{p}$$

where *λ* was thermal conductivity (W/(m·K)), *α* was thermal diffusivity (m^2^/s), *ρ_app_*was the apparent density of the tablet at maximum compression (kg/m^3^), and *C_p_* was the heat capacity (J/kg·K) of the powder. The thermal conductivity die tool was obtained from COMSOL Multiphysics^®^ 5.6 software (provided by CMC Microssytem, Pavillon 1, 3000 boul. de l’Université, Sherbrooke, QC, J1K 0A5, Canada) [57]. Like heat capacity, the thermal conductivity of the punches and die is a function of the local temperature during powder compression.

#### 2.2.3. Powder Retention/Sorption Curve

The isothermal moisture sorption or retention curve of MCC PH101 was obtained by stocking 30 g of powder overnight under 25 ± 2%, 35 ± 2%, 50 ± 2%, 65 ± 2%, 80 ± 2%, and 97 ± 2% relative humidity in a hermetically sealed box. The powder was collected after 12 h, when powder humidity was assumed to have reached an equilibrium state with the surroundings. Powder moisture content was measured one second after it was collected from the box. Three replicates of the powder sample were stored at each relative humidity.

#### 2.2.4. Relative Tablet Density

The relative density of the tablet was calculated with the dimensions of the out-of-die tablet at different rates of axial upper punch pressure:

(4) $${RD}_{calc}=\frac{\rho_{tablet}}{\rho_{particle}}=\frac{m_{tablet}}{V_{tablet}*\rho_{particle}}$$

where *ρ*_*tablet*_ was tablet density (kg/m^3^), *ρ*_*particle*_ was the true powder particle density (kg/m^3^), and $m_{tablet}$ and $V_{tablet}$ were the tablet weight (kg) and volume (m^3^), respectively.

#### 2.2.5. Tableting Process

The powder was compressed with a single-station tablet press (Manesty F3, Federal Equipment Company, Cleveland, OH, USA). The press was operated at a compression speed of 32 mm/s, measured with a proximity sensor (NPN 10 mm, Hall effect 3 wires normally open, 5-24VCC, 200 mA, 320 kHz) coupled with a tachymeter. Compaction pressure was measured with a calibrated load cell (MLC-10K, Transducer Techniques, Temecula, CA, USA) fixed in a loge at the top of the upper punch. The set of punches and die (Natoli Engineering Company, Inc., St. Charles, MI, USA) used were made of ERS S7 steel and A7 steel, respectively, with a diameter of 10.97 ± 0.01 mm and an inner die diameter of 11 ± 0.01 mm.

The maximum compression force of the tablet press was set at 410 MPa. The radial pressure developed on the die wall during compaction was measured indirectly with a linear strain gage (Micro Measurements, A VPG Brand, Raleigh, NC, USA) with a resistance of 350 ± 0.3% ohm and a gage factor of 2.09. The strain gage was fixed at the lower middle of the exterior wall of the die. Radial pressure was calculated from the Hoop stress developed on the exterior surface of the die, which was obtained through the measured strain and Young’s modulus of the die material. The data were acquired with a logger (DI-718B-US, DataQ Instruments, Akron, OH, USA) coupled with amplification modules (8B38) for strain gage and load cell. The sensors’ output was processed with free Windaq software in Windows 10.

#### 2.2.6. Tablet Surface and Peripheral

During compression, the temperature of the top and sides of the tablet was measured one second after its ejection from the die cavity. An FLIR E4 thermal camera (Teledyne FLIR LLC, Wilsonville, OR, USA) with automatic adjustable emissivity was used to detect tablet temperature. The FLIR was manually held to focalize the pointer on the region of interest. The distance between the FLIR and the ejected tablet was approximatively 20 cm, which allowed more precise recording of the temperature, measured for different levels of compaction, i.e., relative densities, for validation with the simulation results. For each compaction level, an average temperature was calculated from 10 samples.

#### 2.2.7. NIR-Penetration Depth

**Figure 2:**
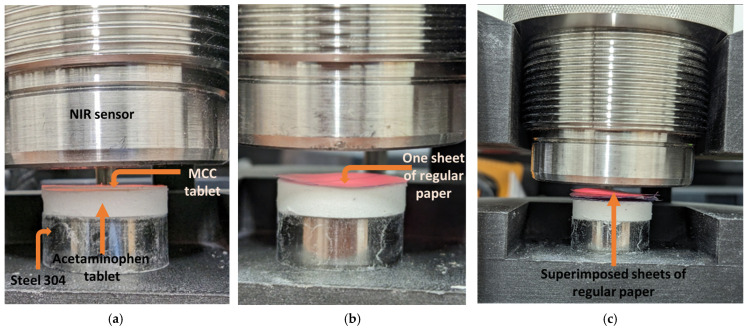
NIR-radiation penetration depth measurement setup. (**a**) MCC disk on acetaminophen tablet, (**b**) one sheet and (**c**) 5 sheets of regular paper on acetaminophen tablet.

To estimate the NIR-radiation penetration depth in the tablet during scanning, an MCC disk and regular paper were placed at the top of an acetaminophen (N-acetyl-para-aminophenol or APAP) tablet. The regular paper disks were used because of their chemical similarity with MCCs and to simulate thinner thickness. All the samples had the same diameter—20 mm. The regular paper has a thickness of 74 microns and porosity of 57%. The MCC disk was obtained from pressed MCC powder at a pressure of 220 MPa and had a thickness of 0.45 mm and a porosity of 38%. The porosity (ε) of the paper and MCC disk was estimated from Equation (4) (ε = 1 − RD_calc_). APAP powder was supplied by Sigma-Aldrich Canada Co. (2149 Winston Park Drive Oakville, ON L6H 6J8, Canada). Figure 2 shows the MCC disk (Figure 2a) and one (Figure 2b) and five paper sheets (Figure 2c) at the top of the APAP tablet.

Primarily, the APAP tablet, MCC disk, and regular paper were scanned individually to register their respective spectra. Then, disks of increasing thickness were stacked at the top of the APAP tablet until the corresponding fingerprint (absorbance from functional group in a specific region) of acetaminophen vanished completely. The thickness at which APAP was not detected was chosen as the maximum penetration depth of NIR radiation.

The regular paper was used at first to find the maximum penetration depth and replaced by the MCC disks to verify the results. The setup for the measurement was maintained to avoid variation in the height at which the sample was scanned. The humidity and temperature in the lab were also controlled.

#### 2.2.8. Determination of Relative Tablet Moisture Content Density

The tablet surface moisture was measured with an NIR sensor in reflection mode at a wavelength of 1100–1700 nm. A calibration model was developed with manually compacted tablets. Then, the tablets pressed with the Manesty single punch were scanned inline to predict the tablet surface moisture content.

To create the calibration model, the powder was spread in an aluminum foil container and equilibrated overnight at 25 ± 2%, 35 ± 2%, 50 ± 2%, 65 ± 2%, 80 ± 2%, and 97 ± 2% relative humidity and room temperature in a humidity-controlled chamber; 30 g of the powder was used for each relative humidity level. After 12 h, the powder was removed from the humidity-controlled chamber, poured into a small, closed plastic box, and stirred manually for 30 s. Powder (3 g) was then sampled from the plastic box for water content measurement, and the rest of the batch was used to manually produce 5 tablets with a lower compaction level (thickness: 6.5 ± 0.8 mm), each weighing 0.5 g. Each tablet was individually scanned five times (25 scans for each relative humidity level). Scans of 30 tablets in total were used for the calibration. The tablets were made in an 11 mm die and pressed with a punch by hand to apply very low pressure, since high pressure can induce a rise in powder particle temperature. All steps in the scan collection phase were performed on the compression plate to mimic the conditions during the tableting process.

The measured water content from the conditioned powder used to make the tablets manually was chosen as a reference value of these tablets’ surface moisture content. The moisture content of the manually produced tablet was considered to be homogeneous throughout the tablet and equal to the moisture content of powder from which it was made. The time between powder sampling from the plastic box and tablet manual compression followed by the scan with NIR sensor was less than 5 s, and hence the moisture content was expected to be preserved. The powder moisture measurement and the tablet manual compression were performed simultaneously. For the calibration model, the moisture content was selected to range between 4.2% and 14%. This range of moisture content was chosen to build a robust model that could predict a high moisture content with minimum error. In fact, the temperature of the tablet was shown to increase during the compression; therefore, it induced the migration of water molecules in the powder bed to the external surface of the tablet.

**Figure 3:**
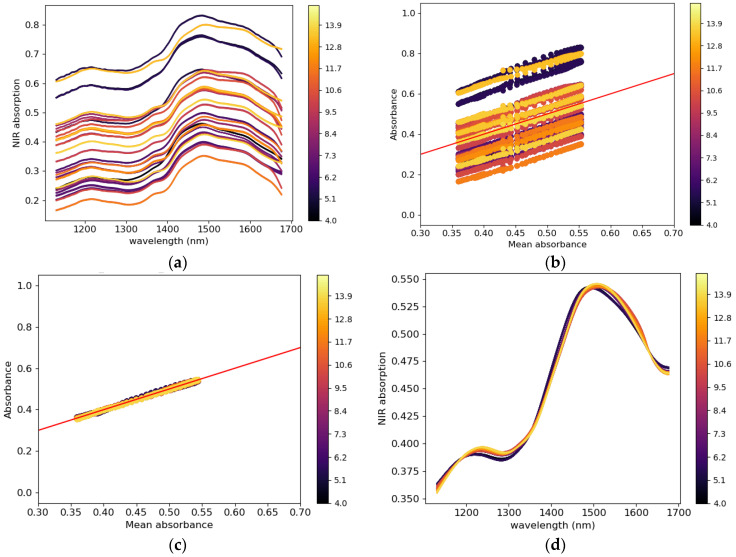
NIR (**a**) raw spectra vs. wavelength, (**b**) individual raw spectra vs. the mean absorbance of all spectra before and (**c**) after pretreatment, and (**d**) spectra after pretreatment vs. wavelength of MCC VIVAPUR PH101 tablets.

The raw spectra collected from the tablet presented noise due to interference and baseline shift due to multiplicative and additive effects caused by the inhomogeneity of the tablet microstructure. All spectra (Figure 3a) are plotted versus the spectral mean, as shown in Figure 3b. The red line represents the diagonal introduced for comparison, the slope of the lines (formed by scatter points) and the intersection with the vertical axis (absorbance) represent the multiplicative and additive effects [58,59]. The lines that have the same slope and same intersection (with the absorbance axis) with the diagonal red have has insignificant multiplicative and additive effects, respectively. in Figure 3b, most lines (scatter points) have the same slope as the diagonal and a non-zero intersection with the absorbance axis. This suggests that some spectra have been affected by the multiplicative effect and most of the spectra are affected by the additive effect. To remove these artefacts, different mathematical preprocess functions, such as multiplicative scatter correction (MSC) and its extended version (EMSC), standard normal variance (SNV), Savitzky–Golay (SG) filter, detrend, and first and second derivative, can be used to treat the spectra [60]. To select the most appropriate preprocessing method for the present data, the pretreated spectra were plotted against the mean spectra and compared with the diagonal line. When the multiplicative and additive effects are removed from the raw spectra, the plotted lines (line formed by scatter points) will align with the diagonal line. The preprocess technic that allows the plotted lines to fit best with the diagonal line was selected as the best-performing technic. In this study, the combination of EMSC and SG (17, 1, 0) was selected, and the results are shown in Figure 3c. The pretreated spectra are shown in Figure 3d.

After pretreatment, Hotelling’s T^2^ in principal component analysis (PCA) [61] with a 95% confidence interval was applied to the data to visualize them and remove outliers. The PLS regression method [62] was used to establish the calibration model with an optimal three principal components. K-fold cross-validation was performed on the data. K-fold cross-validation is an external validation or prediction technic that divides the data into six groups, with each group containing the data of each moisture content level. A calibration model is established with five groups systematically selected and used to predict the sixth group. The process is repeated in a loop until all the groups are predicted by the respective calibration models. At the end of the process, the model performance [54] in calibration and prediction was measured with the regression coefficient (*R*^2^) and root-mean-square error (RMSE) [54]. A mean *R*^2^ and RMSE in calibration and prediction is calculated from the six results.

**Figure 4:**
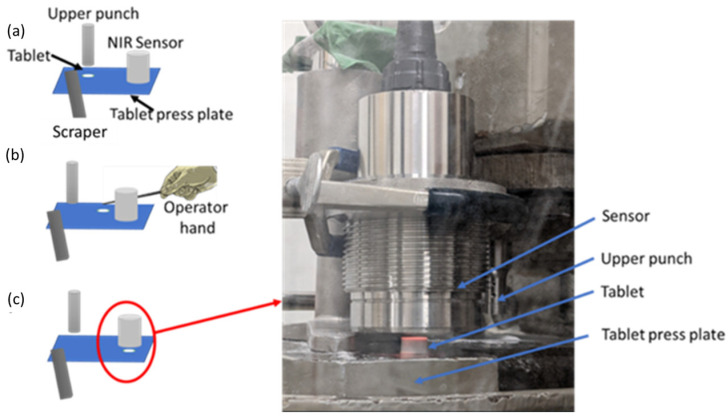
The steps in tablet surface moisture content measurement. (**a**) The tablet is ejected from the die, (**b**) the operator pushed the tablet manually under the NIR sensor and (**c**) the tablet is scanned with the NIR sensor. A video of the measurement can be seen in Supplementary Material S1.

After the calibration model had been computed, the tablets were pressed with compression pressure of 37.5 MPa (thickness of 4.83 ± 0.2 mm) and scanned directly on the tablet press table. Figure 4 shows the steps in the process of inline scan collection on the press. First, the pressed and ejected tablet from the die was pushed by the scraper (Figure 4a) and then the tablet was manually guided (Figure 4b) under the sensor to take measurements (Figure 4c). The entire process took less than 2 s; therefore, the tablet surface moisture was assumed to have been preserved.

#### 2.2.9. Tablet Cohesion and Internal Friction Angle Density

Tablet cohesion and internal friction angle were determined with diametral and axial compression tests. The tablet’s diametral and axial breaking forces were measured with a tablet hardness tester (C50, Whitehall International, New Lane, Havant, UK) and a manual press (Carver Laboratory Press, model 12 Ton, Fred S. Carver Inc., Wabash, IN, USA). Tablets compacted at different relative densities between 40% and 95% were used for the measurements. Tablets with a relative density < 40% were not hard enough for the tests. The hardness tester crushed the tablets along their diametral/radial line and displayed the maximum breaking diametral force (*F_d_*). On the manual press, the tablets were pressed between two horizontal plates until they were crushed. The breaking axial compressive force (*Fc*) was obtained from the pressure applied and the tablet surface. Equations (5) and (6) were used to calculate the breaking radial/diametral tensile strength $\left(\sigma_{d}^{f}\right)$ and axial compressive strength $\left(\sigma_{c}^{f}\right)$ of the tablets [12,16]:

(5) $$\sigma_{d}^{f}=\frac{2F_{d}}{\pi Dt}$$

(6) $$\sigma_{c}^{f}=\frac{{4F}_{c}}{\pi D^{2}}$$

where *D* was tablet diameter (mm) and *t* was tablet thickness (mm).

The breaking force thus determined was used to calculate tablet cohesion (*d*) and internal friction angle (*β*) with Equations (7) and (8) [12,16]:

(7) $$d=\frac{\sigma_{c}^{f}\sigma_{d}^{f}\left(\sqrt{13}-2\right)}{\sigma_{c}^{f}+2\sigma_{d}^{f}}$$

(8) $$\beta =\tan^{-1}[3\frac{\left(\sigma_{c}^{f}+d\right)}{\sigma_{c}^{f}}]$$

## 3. Numerical Method

The aim of the numerical analysis was to simulate the mechanical, heat, and moisture behaviors of the powder bed during the compaction phase. The simulation was based on coupling a solid mechanical module with the heat and moisture transfer in the building materials module implemented in the finite element analysis solver COMSOL Multiphysics^®^.

### 3.1. Simulation Model for the Mechanical Behavior of the Powder

#### 3.1.1. Density-Dependent Drucker–Prager Cap Model

**Figure 5:**
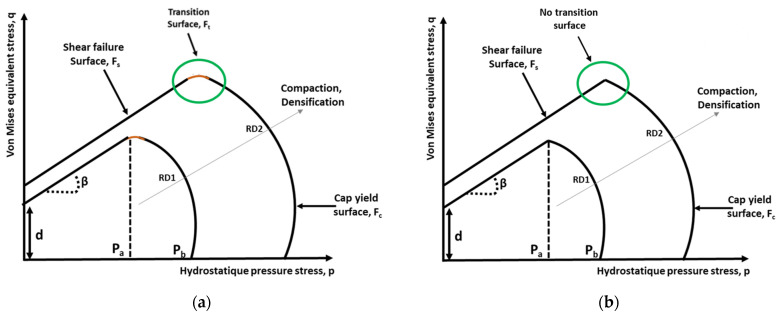
The Drucker–Prager cap model. Yield surface in (p, q) plane: (**a**) from [12]; (**b**) implemented in COMSOL Multiphysics^®^ [63].

The density-dependent DPC model [12] has been used in several studies, which found high agreement between simulated and experimental measurements. The model consists of three surfaces (Figure 5a):

- The Mohr–Coulomb shear failure surface (*F_s_*), representing shear flow, defined as

(9) $$F_{s}=q-p\tan\beta -d$$

- The transition surface (*F_t_*), representing a mathematical smoothing surface, defined as

(10) $$F_{t}=\sqrt{{\left(p-P_{a}\right)}^{2}+{\left[q-(1-\frac{\alpha }{\cos\left(\beta \right)}(\left(d+P_{a}*\tan\beta \right))\right]}^{2}}\;-\alpha \left(d+P_{a}*\tan\beta \right)=0$$

- The cap yield surface (*F_c_*) defined as

(11) $$F_{c}=\sqrt{{\left(p-P_{a}\right)}^{2}+{\left[\frac{Rq}{1+\alpha -\frac{\alpha }{\cos\beta }}\right]}^{2}}-R\left(d+P_{a}*\tan\beta \right)=0$$

where *β* is the material friction angle (°), *d* is its cohesion (Pa), and *p* and *q* are hydrostatic pressure stress (Pa) and von Mises equivalent stress (Pa), respectively, which are expressed as

(12) $$p=\frac{1}{3}(\sigma_{z}+2\sigma_{r})=\frac{I_{1}}{3}$$

(13) $$q=\left|\sigma_{z}-\sigma_{r}\right|=-\sqrt{\frac{3}{2}J_{2}}$$

where *σ_r_* and *σ_z_* are punch stress and die stress, respectively, and *I*_1_ and *J_2_* are the first stress invariant and second deviatoric stress invariant, respectively.

The parameters needed for the elliptic cap are:

- The cap evolution parameter, Pa, that represents the volumetric plastic strain-driven hardening/softening, defined as

(14) $$\begin{array}{c} Pa=-\frac{\left[3q_{B}\;+\;4d\tan\beta *f^{2}\right]}{4\left[{\left(f\right)}^{2}\right]}\\ \;\;\;\;\;\;\;\;\;\;\;\;\;\;\;\;\;\;\;\;\;+\frac{\sqrt{9q_{B}^{2}\;+\;24dq_{B}{\left(f\right)}^{2}\tan\beta \;+\;8\left(3p_{B}q_{B}\;+\;2q_{B}^{2}\right)f2}}{4[(f)2]} \end{array}$$

where *f =* (1 + *α − α*/cos*β*) and *α* is a small number (typically 0.01–0.05) used to define a smooth transition surface between the shear failure surface and the elliptic cap. *P_B_* is the pressure at maximum compression.

- The cap eccentricity, R, is a material parameter between 0.0001 and 1000 that controls the shape of the cap and is defined as

(15) $$R=\sqrt{\frac{2{\left(f\right)}^{2}}{{3q}_{B}}(P_{B}-P_{a})}$$

- The hardening/softening law is a user-defined piecewise linear function relating the hydrostatic compression yield stress, *P_b_*, and the corresponding volumetric inelastic/plastic strain, $\varepsilon_{v}^{p}$:

(16) $$P_{b}=f\left(\varepsilon_{v}^{p}\right)=P_{a}+R(d+P_{a}\tan(\beta ))$$

Volumetric plastic strain can be expressed as $\varepsilon_{v}^{p}=\ln(\frac{RD}{{RD}_{0}})$, where $RD=\frac{\rho_{bulk}}{\rho_{true}}$ is the current relative density and *RD*_0_ is the initial relative density. $\rho_{bulk}$ is the tablet’s apparent density (kg/m^3^) and $\rho_{true}$ is particle true density (kg/m^3^).

#### 3.1.2. Density-Dependent Drucker–Prager Cap Model Implemented in COMSOL Multiphyisics^®^

In the present study, the DPC model version of COMSOL Multiphysics^®^, which uses two yield surfaces, was used [63]. In COMSOL, the Drucker–Prager cone (shear failure surface) always meets the yield cap surface at the point of tangency. Hence, no transition zone is required between the shear failure cone and the elliptical cap, so there is a unique and smooth transition between the two surfaces (Figure 5b). The surfaces were defined based on the first stress invariant I_1_ and the second deviatoric stress invariant *J_2_* as follows.

- The Drucker–Prager yield function, *Fs*:

(17) $$F_{s}=\sqrt{J_{2}}-\alpha^{\prime }I_{1}-k$$

where *α*′ = tan(*β)*/(3√3) and *k* = *d*/(√3) are the Drucker–Prager parameters, *β* is the internal friction angle, and *d* is cohesion.

- The elliptical cap surface, *F_c_*:

(18) $$F_{c}=({\frac{I_{1}-I_{a}}{I_{b}-I_{a}})}^{2}+({\frac{\sqrt{J_{2}}}{J_{a}})}^{2}-1$$

where $J_{a}$ is the ordinate on the $\sqrt{J_{2}}$ axis at $I_{1}=I_{a}.$

Here, the hardening law is defined as:

(19) $${p^{\prime }}_{b}=p_{b0}-K_{iso}\log(1+\frac{\varepsilon_{pvol}}{\varepsilon_{pvol,max}})$$

where *p_b0_* is the initial location of the cap, *K_iso_* is the isotropic hardening modulus, and *ε_pvol,max_* is the maximum volumetric plastic strain.

The cap eccentricity for *R′* i given by:

(20) $$R^{\prime }=\frac{I_{a}-I_{b}}{J_{a}}=\left(3\sqrt{3}\right)*R,$$

where $I_{b}={-3p^{\prime }}_{b}$.

The hardening law in the density-dependent DPC model and that in COMSOL are different; therefore, the parameters *K_iso_* and *ε_pvol,max_* were chosen in such a way that they approximatively fitted the hardening law curve obtained from the calibrated DPC model. The important permanent deformation of the particles during compression was considered and a large plastic strain plasticity model was used. Hence, the relationship between current relative density (*RD*) and volumetric plastic strain is given by:

(21) $$RD={RD}_{0}*J_{P}^{-1}$$

where *J_p_* is the plastic volume ratio.

#### 3.1.3. Nonlinear Elastic Law

The unloading portion of the compression curve of the non-lubricated pharmaceutical powder shows nonlinear behavior, which can be attributed to the dilatation of the tablet during the unloading phase and is described using a nonlinear elasticity law. The density-dependent elastic modules that characterize elastic behavior, such as the bulk modulus *K* and the shear modulus *G*, are expressed in such a way as to avoid path dependence [12]:

(22) $$K=K\left(p,\rho \right)=K(I_{1},\rho )$$

(23) $$G=G\left(q,\rho \right)=G(\sqrt{J_{2}},\rho )$$

where *I_1_* is the first stress invariant, $\sqrt{J_{2}}$ is the stress invariant, and *ρ* is the compact apparent density. When considering the linear portions of the unloading curve, the portion between the maximum compression stress at point B localized on the cap surface and point *C* where the hydrostatic state is reached, i.e., *σ_r_* = *σ_z_*, can be used to determine the elastic modulus using two equations [11].

(24) $$\frac{\sigma_{z}^{B}+\sigma_{z}^{C}}{\varepsilon_{z}^{B}+\varepsilon_{z}^{C}}=K+\frac{4}{3}G$$

(25) $$\frac{q^{B}}{P^{B}+P^{C}}=\frac{2G}{\sqrt{3}K}$$

With the elastic modulus thus determined, the density-dependent Young’s modulus *E* and Poisson’s ratio *ν* can be determined [11,64]:

(26) $$E=\frac{9KG}{3K+G},$$

(27) $$\nu =\frac{3K-2G}{2(3K+G)}$$

### 3.2. Moisture and Heat Transport Simulation Model

During the tableting process, heat is generated due to irreversible deformation and friction phenomena. The heat diffuses through the particles and increases the temperature of the entire powder bed during the compaction phase. The behavior of the inherent moisture of the powder particles remains unknown. Moisture content distribution and evolution can be investigated using the heat and moisture transport inbuilding materials module in COMSOL.

The heat, air, and moisture (HAM) model is widely used for porous materials [65,66] and granular materials [67]. It combines heat transfer, air diffusion, and moisture transport through porous materials. The heat generated diffuses in the powder by a combination of conduction (through the bulk particles) and convection (through interparticle air) and through the tableting tools by conduction. The powder bed is a granular medium with interparticle pores. Moisture can exist in pores in two thermodynamic states of matter—vapor and liquid [66]. The main mechanisms associated to moisture transfer are vapor diffusion or capillary suction, or both. The material has a unique equilibrium moisture content characteristic curve that covers the hygroscopic and capillary water regions. These regions are commonly referred to as sorption isotherm and retention curves. Inside the hygroscopic region, the pores are mainly filled with water vapor, and consequently, moisture transport is mainly achieved by vapor diffusion. Liquid water transport is possible when pores are filled with liquid water. This flow mechanism is very active in the capillary water region, where the relative humidity is over 95%. Both vapor and liquid transport can coexist at the higher end of the hygroscopic region, where vapor diffusion and capillary suction are active in large and small pores, respectively. Water vapor diffuses into open pores and condenses on the capillary meniscus, whereas water evaporates into the next open pore space at the other end of the meniscus. This implies that the diffusion path is reduced, thereby increasing the moisture evaporation rate.

The moisture balance can be expressed using several thermodynamic potentials dependent on transport–diffusion partial differential equations. Relative humidity and temperature are the dependent variables in the mathematical model. The equation that governs moisture transfer is as follows [57,66]:

(28) $$\frac{\xi \partial \varphi }{\partial t}+\nabla \cdot \left[\xi D_{w}\nabla \varphi -\delta_{p}\nabla \left(\varphi p_{sat}\left(T\right)\right)\right]=G$$

It represents the sum of vapor diffusion flux and capillary moisture flux. Their respective representative equations are:

(29) $$g_{1}=-\delta_{p}\nabla \left(\varphi p_{sat}\left(T\right)\right)$$

(30) $$g_{2}=\xi D_{w}\nabla \varphi$$

where φ is relative humidity (%), *δ_p_* is vapor permeability (m^2^/s), *p_sat_* is saturation pressure (Pa), *w* is moisture capacity (kg/kg), *D_w_* is moisture diffusivity (m/s^2^), *G* is the generic moisture source (kg/m^3^), and *T* is temperature (K).

The moisture source is water molecules adsorbed on the powder surface and pores that evaporated during the tableting process. The coupled thermal analysis is solved by the following form of the energy balance equation [20,66]:

(31) $${\left(\rho C_{p}\right)}_{eff}\frac{\partial T}{\partial t}+\nabla \cdot (-\lambda_{eff}\nabla T-{L_{v}\delta }_{p}\nabla \left(\varphi p_{sat}\left(T\right)\right))=Q$$

where *ρ* is effective density (kg/m^3^), *C_p_* is specific heat at constant pressure (J/(kg·K)), *λ* is thermal conductivity (W/(m·K)), *L_v_* (J/kg) is heat of evaporation, and *Q* is the heat source (W/m^3^). The energy balance is applied under the assumption of local thermodynamic equilibrium (LTE). In the first term of Equation (31), effective thermal capacity is defined as follows [20]:

(32) $${\left(\rho C_{p}\right)}_{eff}=\rho_{s}C_{p,s}+wC_{p,w}$$

where ${\left(\rho C_{p}\right)}_{eff}$ is effective volumetric heat capacity at constant pressure, defined to account for both solid matrix and moisture properties; $C_{p,s}$ and $\rho_{s}$ are the density and specific heat capacity of the dry solid; *w* is water content given by the moisture storage function, i.e., the moisture sorption curve for the chosen material; $C_{p,w}$ is water heat capacity at constant pressure; and $\lambda_{eff}$ is the effective thermal conductivity function of the solid matrix and moisture properties [20,66]:

(33) $$\lambda_{eff}=\lambda_{s}(1+\frac{bw}{\rho_{s}})$$

where $\lambda_{s}$ is the thermal conductivity W/(m·K) of the dry solid and *b* (dimensionless) is a thermal conductivity supplement due to the water content. Equation (31) is composed of two heat sources: sensible heat due to moisture content variation in the powder, expressed as vapor diffusion flow multiplied by latent heat of evaporation ($L_{v}$); and the heat source caused by irreversible deformation, labeled as *Q*. The heat source *Q* relates to plastic strains, stress, interparticle friction, and friction with die walls, and is given as [18,20]:

(34) $$Q=q_{1}+q_{2}$$

where $q_{1}$ is the heat generated by interparticle friction and permanent deformation, and $q_{2}$ is the heat generated by particle–wall friction. Ninety percent of the energy needed to permanently deform the particles is assumed to dissipate as heat in the powder bed [18,20]. $q_{1}$ is defined as

(35) $$q_{1}=\xi \sum_{i,j=1}^{3}\sigma_{ij}{d\varepsilon }_{ij}^{pl}$$

where *ξ* = 0.9 represents the irreversibly deformed heat fraction [18,20].

The heat generated by friction is defined as

(36) $$q_{2}={\upsilon \mu ʃ}_{A}\sigma_{nn}dA$$

where *μ* is the wall friction coefficient (-), *A* represents the interacting area between the powder and die wall (m^2^), *σ_nn_* is the local normal stress (Pa), and *υ* is the norm of the local slip velocity at the interface between the powder and the die (m/s).

Friction heat is an interface heat between the die wall and the powder compact. The portion of heat transferred to the powder compact can be calculated with the weighting factor proposed in [68,69,70] and adopted by [20]. Hence, friction heat can be separated into two parts:

(37) $$q_{2die}=\eta *q_{2},$$

(38) $$q_{2tablet}=\left(1-Ƞ\right)*q_{2},\;with\;\theta =\left(1-Ƞ\right)$$

where Ƞ is the weighting factor, which is estimated by Equation (39):

(39) $$Ƞ=\frac{1}{1+\left(\frac{3*\lambda_{powder}}{2*\lambda_{die}}\right)*\sqrt{\frac{\alpha_{die}}{\alpha_{powder}}}},$$

where *λ_powder_*, *λ_die_*, *α_die_*, and *α_powder_* are powder and die thermal conductivities and powder and die thermal diffusivities, respectively.

### 3.3. Method of Calculation of Elastic Parameters and Drucker–Prager Cap Model Parameters

The calibration procedure used in this study to determine the DPC model parameters and elastic parameters is described by [12]. Elastic parameters, such as Young’s modulus and Poisson coefficient, were determined from the unloading curve. The DPC model parameters, cohesion (*d*) and internal friction angle (*β*) were first obtained with diametral and axial compressive tests, then cap eccentricity (*R*) and hydrostatic compression yield stress (*P_b_*) were calculated with the *d* and *β* obtained.

### 3.4. Finite Element Model

**Figure 6:**
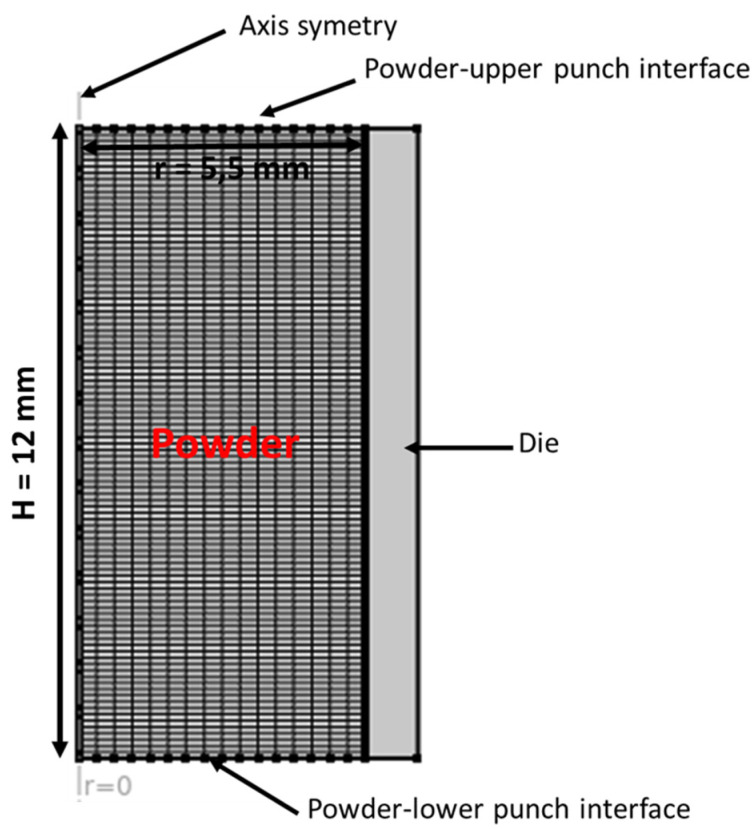
The finite element model of die compaction using flat-faced punches.

The FEM simulations of the compaction phase of the tableting process were performed on the commercial COMSOL Multiphysics^®^ 5.6 finite element analysis solver, which implemented the DPC plasticity model and HAM model. MCC VIVAPUR PH101 was used as the model powder. A flat-faced, cylindrical compact geometry was modeled with the compact/powder represented by a four-node, 2D axisymmetric model, as shown in Figure 6. The powder was modeled as continuum, isotropic, poroplastic, and deformable media in which the pores were uniformly distributed. The flat-faced upper/lower punches and the cylindrical die were modeled as analytical rigid bodies without any deformation. The slip velocity boundary condition between the powder and the die wall was set to simulate the rate at which compression occurred. The finite element simulations were modeled as quasi-static; therefore, punch velocity and dwell time were disregarded. Only the upper punch was set to move downward to simulate one side compression. The initial powder fill height was 12 mm in a die with a radius of 5.5 mm, and the powder was compacted with the upper punch to a final height of 4.5 mm during the compaction stage. In the analysis, only the compaction phase of the quasi-static compaction process was simulated. The wall friction effect was considered through the adoption of a Coulombic boundary condition at the powder–die wall and powder–punch interfaces. Since MCC PH101 was not lubricated, a static Coulombic friction of 0.21 was adopted from [12].

Our simulations used a constant cap eccentricity (R′) and a density-dependent constitutive model parameter (cohesion [d], internal friction angle [β], Drucker–Prager parameters [α′, k], or hydrostatic yield compression stress [P_b_′]). The mechanical properties of the powder as a function of relative density were expressed in the range of 40% to 80% compaction and are summarized in Supplementary Material S2. A piecewise function was used in COMSOL Multiphysics^®^ to implement all mechanical parameters.

**Figure 7:**
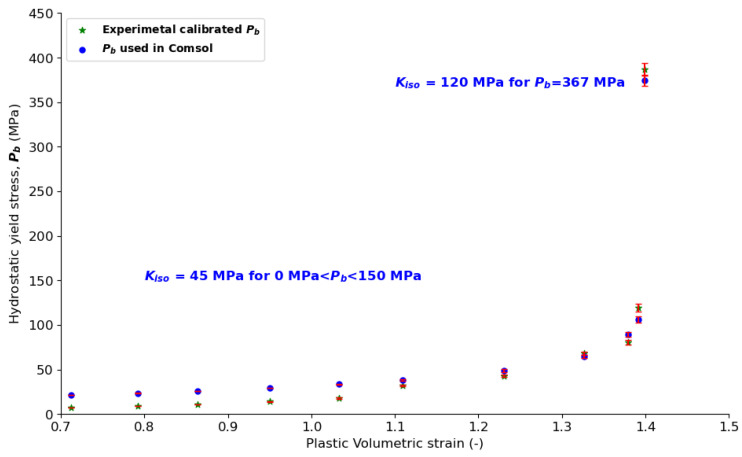
Hydrostatic yield stress, P_b_ vs. plastic volumetric strain.

Yield compression stress (P_b_′), i.e., the hardening law, and cap eccentricity (R′) in the COMSOL DPC model were calculated with Equations (20) and (21), respectively. The isotropic hardening modulus K_iso_ was determined (a) to relatively approximate the experimental calibrated DPC model’s hydrostatic yield compression stress (P_b_) (Figure 7); and (b) to ensure consistent powder hardening during the compaction phase. The point at P_b_ = 367 MPa was separated from the rest of the points (Figure 7) because the powder’s incompressible state was reached. For P_b_ lower than 367 MPa, relatively good agreement was reached between the experimentally calibrated points and the calculated points.

**Figure 8:**
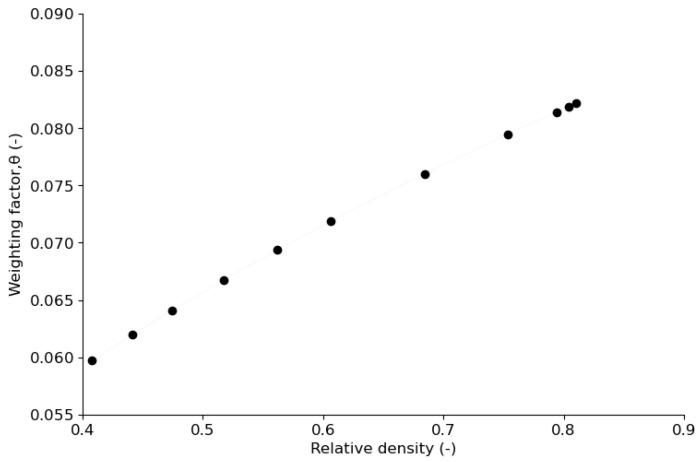
Change in weighting factor, θ, during compaction.

The heat was set to dissipate from the system through a heat flux at the interface between the die wall and the upper and lower punches. At the powder–die wall interface, a boundary heat source was set with a weighting factor function of the relative density of the powder bed. The weighting factor was calculated with Equation (39), assuming that the thermal conductivity coefficient of the powder bed and the thermal conductivity and diffusivity of the die were constant during compaction. From the weighting factor obtained, the portion (θ) of the interfacial frictional heat that transfers to the powder compact by conduction at each level of compaction is shown in Figure 8. As the relative density of the powder bed increases, the portion of heat that diffuses into the powder bed also increases. The thermal diffusivity, $\alpha_{powder}=\frac{\lambda_{powder}}{{\rho C}_{p}}$, of the powder bed (where *λ_powder_*, *ρ*, and *C_p_* are thermal conductivity, apparent density, and heat capacity, respectively, of the powder bed) decreases when apparent density increases. Hence, the weighting factor, Ƞ, which is proportional to the square root of the thermal diffusivity of the powder bed, decreases during compaction and θ increases (Figure 8). The fraction of irreversible work converted into heat has been estimated to be in the range of 80–100% [20,69]. Hence, in this study, 20% of the input mechanical energy inside the powder bed was assumed to be stored as irreversible particle deformation and 80% was assumed to be converted into heat. The thermal expansion coefficient, $\alpha_{T}=1.0\times 10^{-4}\;[{}^{\circ}C^{-1}]$, of the powder was adopted from [18].

The coefficient, U, for heat transfer between the compression tools and powder compact was determined with the thermal resistance method [71]. The heat transfer between the powder and punch, U_powder-die_, and between the powder and die wall, U_powder-punch_, were equal to 32 W/(m^2^.K) and 18 W/(m^2^.K), respectively (See Supplementary Material S3).

In the proposed model, the moisture was set to flow toward the external environment from the powder compact through the upper punch interface. The powder compact–die wall and powder compact–lower punch interfaces were considered closed, so no moisture could evaporate from those interfaces to the surrounding environment. The experimental powder moisture sorption or moisture retention curve was used in the model, as the moisture content of the powder bed is a function of the relative humidity of the powder during compaction. The moisture transport coefficients of MCC Vivapur PH101 used in the HAM model are given in our recent article [72].

The initial temperature of the powder was set to ambient—21.4 ± 0.2 °C. The moisture content of the powder was set at 4.5%.

## 4. Results and Discussion

### 4.1. NIR Penetration Depth

**Figure 9:**
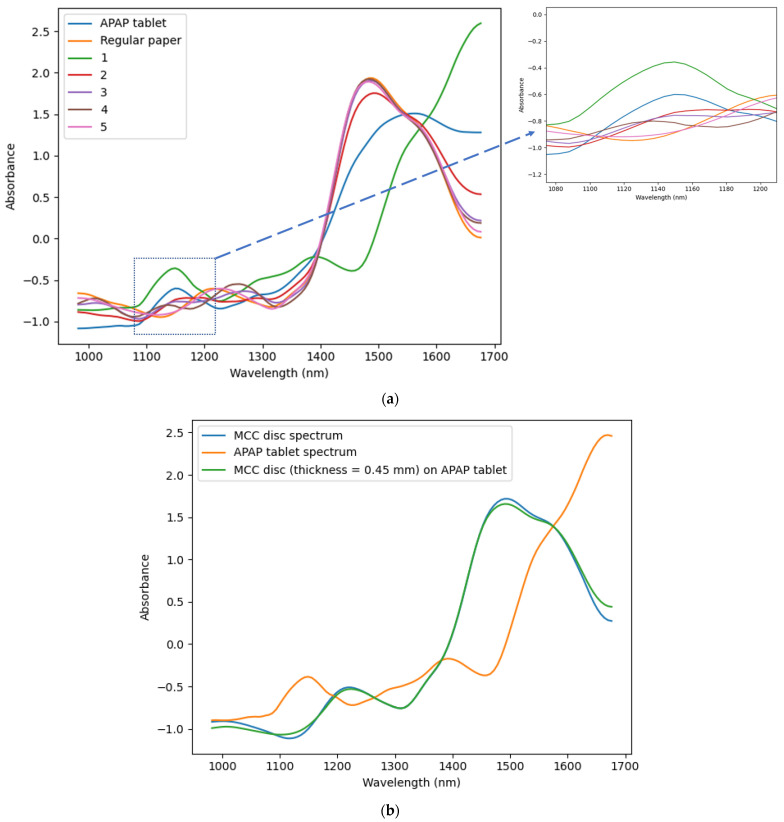
SNV + SG (17, 2, 0) transformed NIR spectra obtained from (**a**) APAP, paper sheet and paper sheet superimposed on APAP tablet surface, (**b**) APAP, MCC disk and MCC disk with 0.45 mm of thickness superimposed on APAP tablet surface. 1: one sheet (thickness = 74 μm), 2: two sheets (thickness = 148 μm), three sheets (thickness = 222 μm), four sheets (thickness = 296 μm), five sheets (thickness = 370 μm) of regular paper stacked on APAP tablet surface.

This work aimed to study the surface moisture of pharmaceutical tablets; therefore, the penetration depth of the NIR radiation into a tablet was estimated. The NIR sensor was used in diffuse reflectance measurement mode. Figure 9 shows the SNV + SG (17, 2, 0) transformed NIR spectra of the APAP tablet, MCC disk, regular paper, and NIR spectra of the MCC disk and sheet of regular paper superimposed on the APAP tablet surface. Pretreatment was applied to remove additive and multiplicative effects and noise from the spectra. The aim of adding several thin layers at the top of the APAP tablet was to determine the critical thickness at which the APAP tablet cannot be detected by NIR radiation.

APAP absorbed NIR radiation at a wavelength of 1135 nm due to the C-H stretching second overtone in the aromatic ring [73]. Cellulose (MCC and regular paper) absorbed NIR radiation at 1220 nm due to hydrogen bond bending and at 1450 nm due to the first overtone of O-H stretching [73]. The absorption peak of APAP at 1135 nm vanished when five sheets of regular paper was superimposed at the top of APAP tablet, which corresponds to a thickness of 0.33 mm or 330 μm (Figure 9a, pink spectrum). Therefore, the NIR-radiation maximum penetration depth can be estimated to be less than 330 μm when a pressed cellulose such as regular paper is scanned. To confirm the results, an MCC disk of 0.45 mm (38% of porosity and water content of 4.25%) was placed at the top of the APAP tablet and the APAP absorption peak at 1135 nm was not detected (Figure 9b, green spectrum). The small air gap between the APAP tablet and the paper sheets or the MCC disk did not affect the NIR signal because the tests were done in dry air (RH = 27.4%).

The increase in moisture content in granular material was found to decrease the reflectance intensity of NIR light [74], because as the moisture content increases, more light is absorbed by the water molecules. The absorption of the incident NIR light by the water molecules at the surface of the scanned sample reduces its incident intensity, thereby decreasing its penetration depth into the sample according to the Beer–Lambert law [75]. Based on these results, the use of NIR in diffuse reflectance mode in this study can be considered to penetrate less than 450 μm into the MCC tablet, and the penetration depth will further decrease as the surface moisture content increases. Hence, only the tablet surface moisture will be captured during the analysis.

### 4.2. Tablet Compression Curve

**Figure 10:**
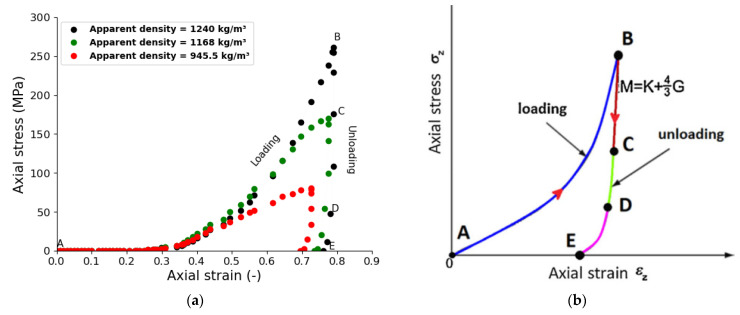
(**a**) Axial stress–axial strain compaction curve of the microcrystalline cellulose (MCC), VIVAPUR PH101. (**b**) Typical axial stress–axial strain curve of MCC. (M is the compression modulus; G is the tablet shear modulus and K is the bulk modulus). A-B: Loading phase; B-E: Unloading phase; B-C: Linear elastic, C-D and D-E non-linear elastic behaviour of the compact.

The compression stress–strain curve obtained for MCC VIVAPUR PH101 in this study at different densities of compaction is shown in Figure 10a. The curve obtained is characteristic of the behavior of the MCC powder under pressure (Figure 10b) [12,16]. The stress–strain curve can be split into two parts—the loading and unloading phases. The loading phase, line AB, describes the volume reduction of the powder bed. During volume reduction, two main phenomena take place for soft particles such as MCC—the escape of excess air from the powder bed interstices and densification. The latter can be explained by three complex mechanisms [16,76]. The first mechanism takes place at the early stage of compaction, during which the particles rearrange due to their inherent movement. On contact, the tangential and normal components of the contact force between particles take part in the organization and elastic–plastic deformation of the particles, respectively. The second mechanism is the elastic–plastic deformation that occurs due to contact interactions between neighboring particles, leading to hardening. The third mechanism is described by the sharp increase in material flow resistance due to material strain hardening. The unloading phase, line BE in Figure 10, is where the upper punch is retracted, and the tablet tends to revert to expand due to its elastic recovery. During the first step, line BD, the material tends to release excess energy stored in the compact that does not participate in the microstructure of the compact. At the end of the unloading phase, line DE, the powder exhibits strong nonlinear behavior due to dilatation of the particles in the compact. The stress–strain curve obtained is consistent with previous results [77].

### 4.3. Mechanical, Heat, and Moisture Simulation during Compaction

#### 4.3.1. Relative Density Evolution and Distribution

**Figure 11:**
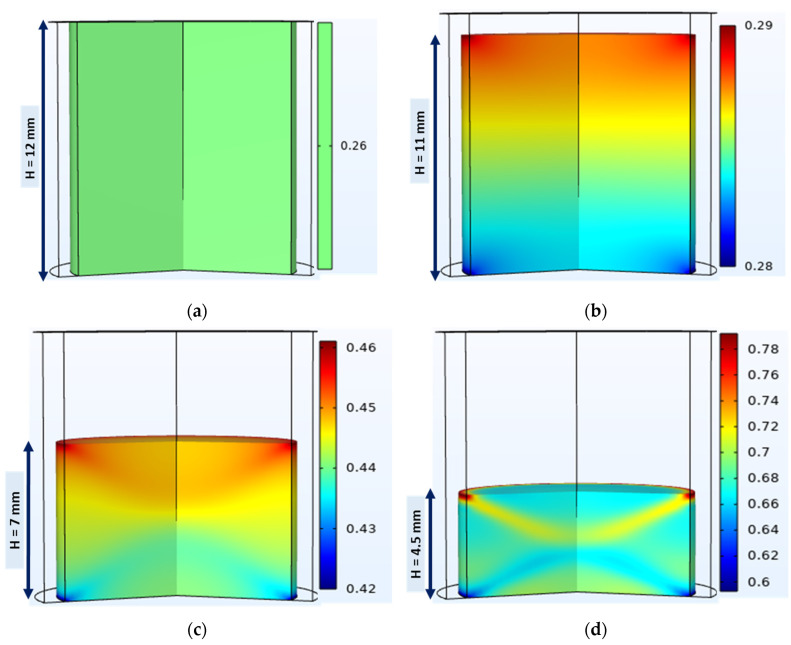
A 3D view of the simulated relative density evolution and distribution during compaction of a model powder (MCC VIVAPUR PH101) with flat-faced punches. The compaction process progresses from (**a**–**d**).

Mechanical simulation of the compaction phase of the tableting process resulted in the evolution of the average relative density of the compact and its distribution on the 3D volume of the die. Figure 11a shows the onset of compaction with a uniform relative powder density of 0.26. During compaction, the upper edges of the powder bed were denser than the rest of the powder bed and the lower edges were the least dense (Figure 11b–d); similar results were obtained in other studies [20,29,41]. This can be explained by die wall friction, which induces a stress gradient inside the powder bed [38] and prevents the powder from sliding along the interfaces [78]. At the end of the compaction, the relative density of the powder was somewhat uniformly distributed, and the top-right corner of the tablet showed a relatively denser region, which was confirmed with X-ray tomography analysis [27,79]. This highly dense region was found to be the point of origin of the crack that initiated tablet lamination [27,79].

**Figure 12:**
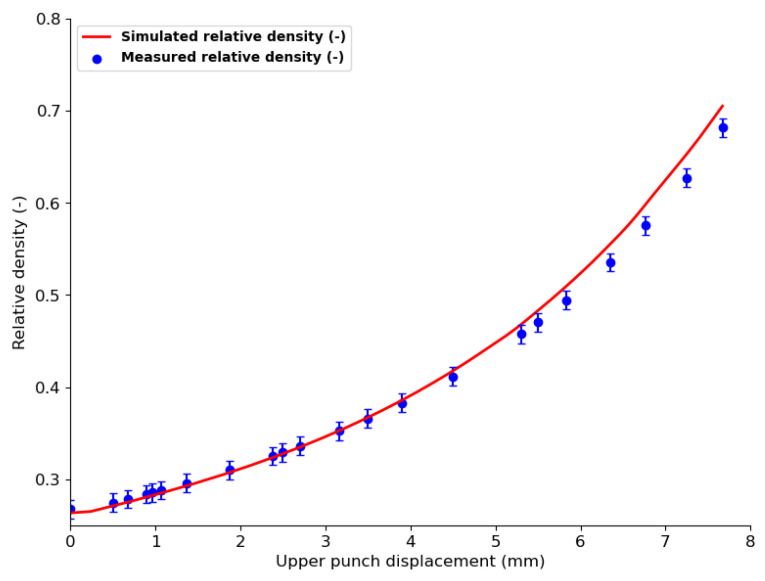
Comparison between the in-die tablet’s simulated relative density and the ejected tablet’s relative density evolution during compaction of MCC VIVAPUR PH101 powder with flat-faced punches.

To validate the mechanical simulation, the average relative density measured for the out-of-die tablet and the relative density obtained from the simulation were compared (Figure 12). The variation in relative density of the tablet with upper punch displacement is presented. Good agreement between simulated and measured results was observed for lower compaction, i.e., low upper punch penetration. For higher compaction i.e., high upper punch penetration (from 4 mm), the simulated relative density was slightly higher than the measured values. These observations are due to the fact that at lower punch penetration, i.e., lower compaction force, particle deformation is not high enough to cause high relaxation of the out-of-die tablet; hence the density calculated from tablet dimensions is relatively close to that obtained for the in-die tablet in the simulation. At higher penetration, i.e., higher compaction force, particle deformation becomes important such that relaxation of the out-of-die tablet also becomes more important, highly impacting the out-of-die tablet dimensions and consequently its relative density.

#### 4.3.2. Temperature Evolution and Distribution

- Simulated temperature of tablets

**Figure 13:**
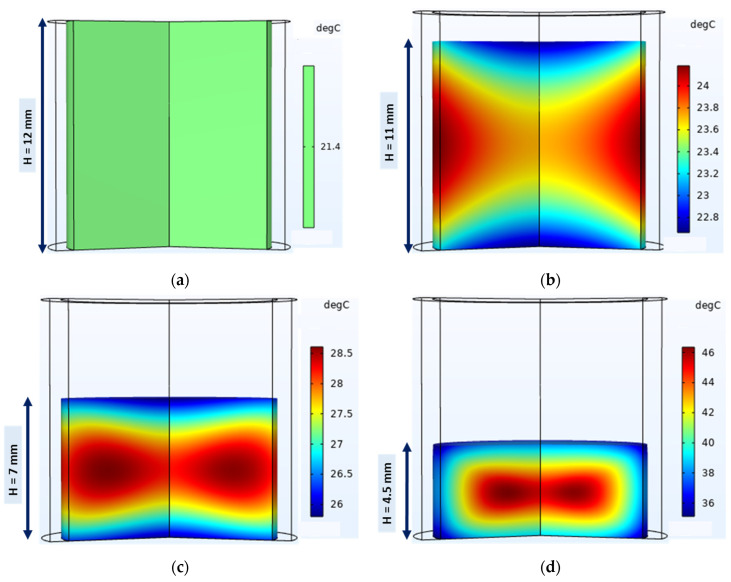
A 3D view of the simulated temperature evolution and distribution during compaction of a model MCC PH101 VIVAPUR powder with flat-faced punches. The compaction process progresses from (**a**–**d**).

The evolution of the 3D powder bed temperature and its distribution during compaction are shown in Figure 13a–d. At the beginning of the compaction phase (Figure 13b), the temperature increases from 21.4 °C (initial loose powder temperature) to around 24.5 °C at the powder bed–die wall interface and around 22.4 °C at the surface in contact with the punches. As compaction continues (Figure 13c), the temperature continues to increase and the temperature at the punch–powder bed interfaces remains lower than the temperatures at the center of the powder bed and at the die wall–powder bed interface. At the end of compaction (Figure 13d), the same temperature distribution was observed in all interfaces, i.e., punch–tablet and die wall–tablet (about 40 °C), but the temperature at the center of the tablet remained higher (about 46 °C). However, the edge of the tablet showed the lowest temperature (about 36 °C). The simulation of the entire compaction phase showed that heat was generated mostly at the tablet–die wall interface and the center of the tablet, as shown in other simulations [18,20]. The heat generated in the tablet flowed from the center to the outer layers by conduction within the tablet and was dissipated from the tablet by conduction through the die wall and punches. The low temperature at the edges of the tablet may be correlated with the higher density of these regions (Figure 11). High density induces higher thermal effusivity at the edges of the tablet and thus lower temperatures in these regions. The overall temperature distribution in the tablet at the end of compaction was consistent with previous studies [18,20,80].

- Measured surface and peripheral temperatures of the tablets

**Figure 14:**
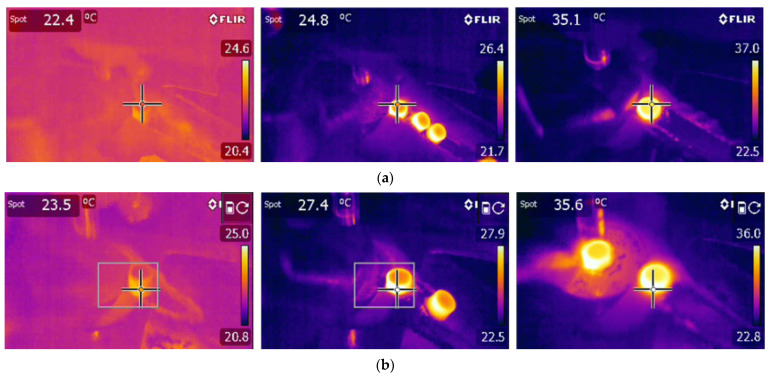
Thermal infrared camera images of (**a**) the tablet surface and (**b**) tablet periphery showing temperature distribution and evolution during compaction of MCC VIVAPUR PH101 powder with flat-faced punches. The relative density increases from left to right (RD1 = 0.26%, RD2 = 0.5%, and RD3 = 0.75%) pressed with a compression pressure of 80 ± 3.5 KPa, 18.8 ± 1.3 MPa and 68.5 ± 5.4 MPa, respectively.

The out-of-die tablet surface temperature and peripheral temperature were measured with a thermal infrared camera one second after ejection. Figure 14 shows the thermal infrared camera images of the compacted tablet at increasing relative density. The images show a clear increase in temperature during compaction. The rise in temperature is explained by the heat generated by the irreversible deformation of powder particles, interparticle friction, and particle–die wall friction [20,77,81]. Furthermore, the out-of-die tablet’s surface temperature was lower than its peripheral temperature. The higher temperature at the periphery may be due to the additional heat generated at the tablet–die wall interface through friction during the ejection phase [18]. By increasing the level of compaction of the powder bed, the temperature of all parts of the tablet increased because more friction and irreversible deformation phenomena occurred as the tableting pressure increased. Similar results were obtained by other studies [18,77].

**Figure 15:**
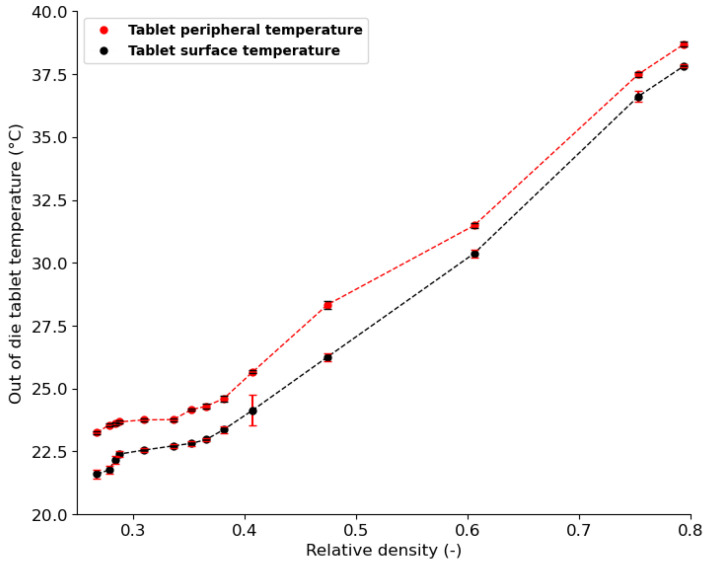
Temperature evolution measured during compaction of MCC PH101 VIVAPUR powder with flat-faced punches.

**Figure 16:**
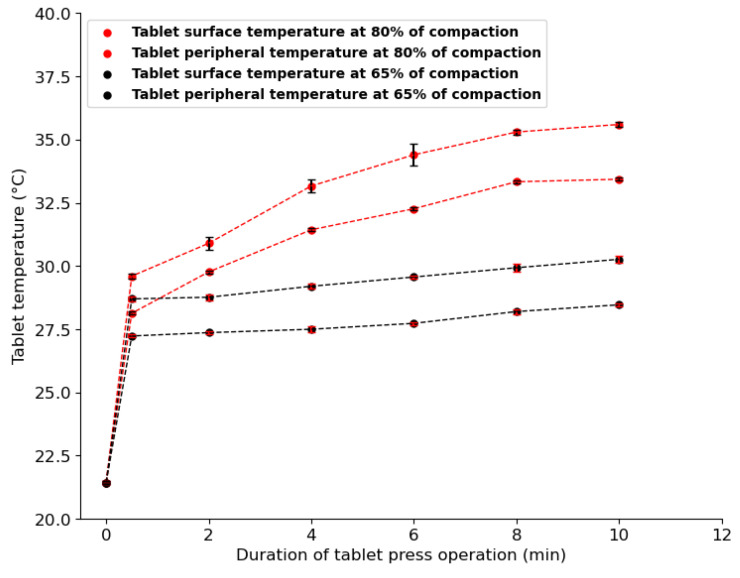
Temperature evolution of MCC PH101 VIVAPUR tablets as a function of the duration of tablet press operation. The tablets were made by compressing MCC powder with flat-faced punches. 600 tablets were produced in 10 min.

To investigate the temperature evolution during a single compaction phase, the temperatures of out-of-die tablets obtained at increasing levels of compaction, i.e., increasing relative density, were measured. Figure 15 shows the temperature evolution with increasing relative density. Both peripheral and surface temperatures increased with increasing compaction. As the level of compaction increases, the contact pressure between particles increases [76,81]; hence, more heat is generated and causes the temperature in parts of the tablet to increase. The peripheral tablet temperature is higher than the surface temperature at all levels of compaction, which may be explained by (1) the additional frictional heat generated at the tablet–die wall interface during ejection and (2) the difference in the rate of heat loss between the surface and periphery of the tablet. The temperature evolution curve can be separated into two distinctive phases. (1) A low-compression pressure phase (21.4% < relative density < 35%) in which a small temperature change, from 21.4 °C to 24 °C, is observed. During this phase, particle reorganization and interparticle friction dominate [16], which may explain the low rise in temperature. (2) A high-compression pressure phase (35% < relative density < 80%) with a high temperature change, from 24 °C to 38 °C, is observed. During this phase, particles are close enough to deform irreversibly on contact and the consolidation between particles induces more radial pressure, which may cause stronger wall friction [76,81]. Therefore, a higher temperature change is observed during the second phase. These two phases observed during temperature evolution were consistent with the loading phase curve (Figure 10a), where axial pressure is low in the region of 21.4% < relative density < 35%, followed by a sharp increase in pressure from 35% to about 80% relative density. Furthermore, as the number of tableting runs increased, tablet temperature increased gradually (Figure 16). As the number of compressions runs increases, compression tools become hotter and transfer additional heat to powder particles, leading to gradual temperature rise [77,80].

**Figure 17:**
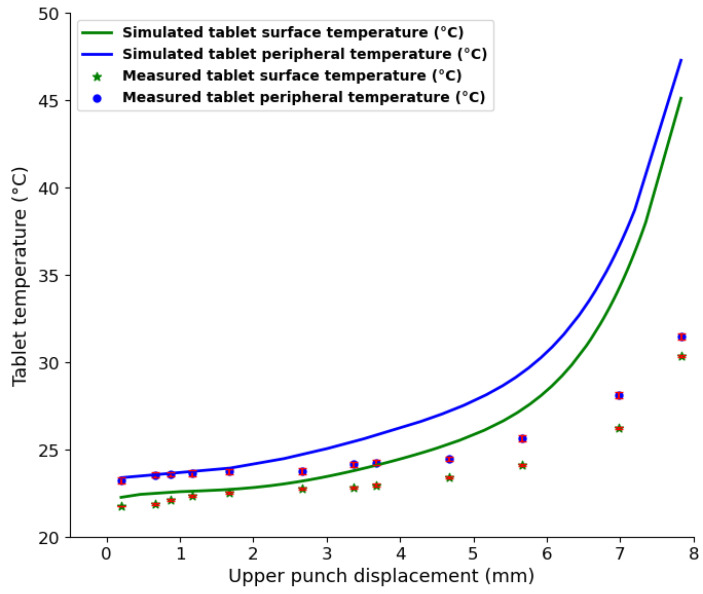
The evolution of the measured and simulated temperatures of MCC PH101 VIVAPUR tablets as a function of upper punch penetration.

The simulation results were compared to the measured temperature for validation purposes. Figure 17 shows the measured temperature of the ejected tablet compared to the simulated temperature of the in-die tablet as a function of the level of compaction, which is represented by punch displacement in the die. The measured and simulated temperatures increased with the level of compaction. In the cases of both measured and simulated temperatures, the tablet’s peripheral temperature was higher than its surface temperature. In the simulation results, the difference in temperature between the surface and the periphery may be explained by the rate of heat generation and dissipation at each interface during compaction. In the experimental results, the additional heat generated during ejection may be responsible for the difference in temperature.

#### 4.3.3. Tablet Moisture Content Evolution and Distribution during Compaction

At low upper punch penetration (upper punch displacement of <2 mm), the simulated results and the measured results matched. However, as the punch went deeper into the die, the simulated temperature of the in-die tablets became gradually higher than the measured temperature of the ejected tablets. Because the measured temperatures were obtained from ejected tablets, during the ejection phase, water molecules could have evaporated from the tablet surface before the measurements were taken. The latent heat used to evaporate the water molecules could have produced a cooling effect on the tablet. For instance, at maximum punch penetration (about 7.83 mm in the die), the difference between the average simulated temperature and the average measured temperature was about 15 °C. Assuming that water evaporated from the top 2 mm of the tablet, the corresponding amount of water evaporated is about 1 mg. With the initial amount of moisture being 43 mg of water per 1 g of powder, the evaporation of 1 mg during tablet ejection is plausible. The agreement between the simulated and measured temperatures at low punch penetration distance may be explained by the low water evaporation rate due to the low temperature rise. A decrease in tablet temperature during ejection was also found in other studies [18,77].

**Figure 18:**
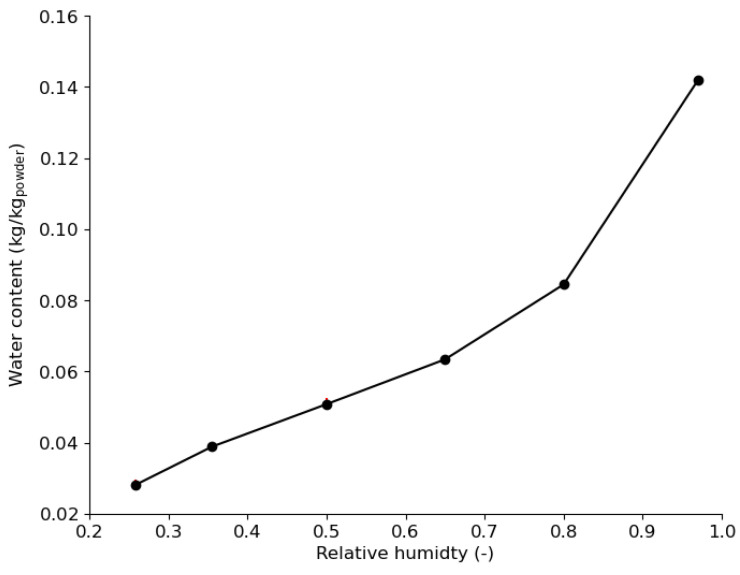
The sorption curve of MCC VIVAPUR PH101 powder.

Figure 18 shows the moisture sorption isotherm curve of the MCC VIVAPUR PH101 used in this study. The curve has a type II and/or III shape according to the Brunauer classification [82], which is obtained for highly porous material, such as the MCC PH101 used in this study, which is well known to have high intraparticle porosity. The type II shape indicates that adsorption begins with the first monolayer between the powder and water molecules at lower humidity, followed by multilayer adsorption between water molecules, and finally leading to capillary condensation at higher humidity [47]. The moisture sorption curve was used in COMSOL Multiphysics^®^ software to follow the water content in the powder bed as the compaction level increased.

**Figure 19:**
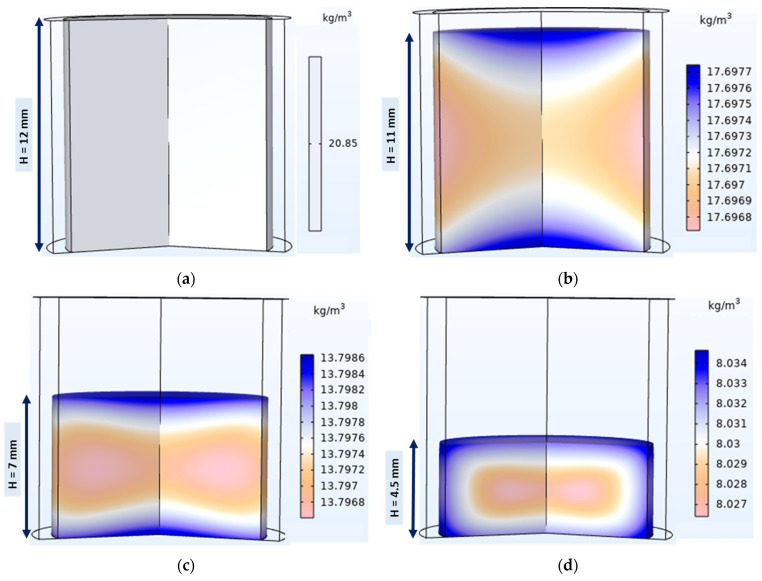
A 3D view of the simulated moisture content evolution and distribution during compaction of model powder (MCC VIVAPUR PH101) with flat-faced punches. The compaction process progresses from (**a**–**d**).

Figure 19 shows the moisture distribution in the tablet during the compaction phase. During compaction, the heat generated can be transmitted as thermal energy to water molecules in the form of kinetic energy. The water molecules that have enough kinetic energy can diffuse between particles and intraparticle pores in the powder bed [27,72,83] due to the water vapor pressure gradient between the powder bed and the surrounding environment. The air contained in the powder bed diffuses radially from the center to the periphery of the tablet and escapes from the powder bed at the edges of the tablets [27] during compaction. The outflowing air carrying water vapor largely contributes to the path of water vapor diffusion. Consequently, the water content of the tablet is higher at the periphery than at the center (Figure 19b–d). Furthermore, the higher temperature of the center of the tablet can lead to higher kinetic energy in the water molecules in this region. Hence, the high heat coupled with the air path may explain the lower water content in the center of the tablet (Figure 19d). The tablet edges appeared moister because of the higher powder density, which can slow down the rate of diffusion of water molecules to the surrounding environment.

During compaction, the overall water content density of the powder bed decreased from 20.85 kg of water/m^3^ of powder to about 8 kg of water/m^3^ of powder. The decrease in water content may be explained by the evaporation of water molecules from the powder compact or tablet to the surrounding environment, particularly the interfaces. Because the simulation in this study allowed water molecules to evaporate from the tablet surface, no accumulation was observed on the tablet surface.

**Figure 20:**
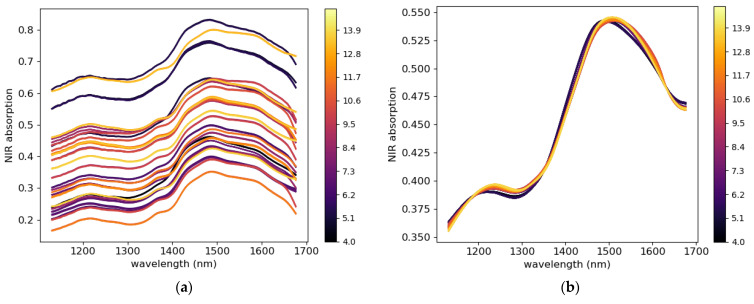
(**a**) Raw near-infrared (NIR) and (**b**) pretreated NIR spectra collected on the surface of MCC VIVAPUR PH101 tablets.

**Figure 21:**
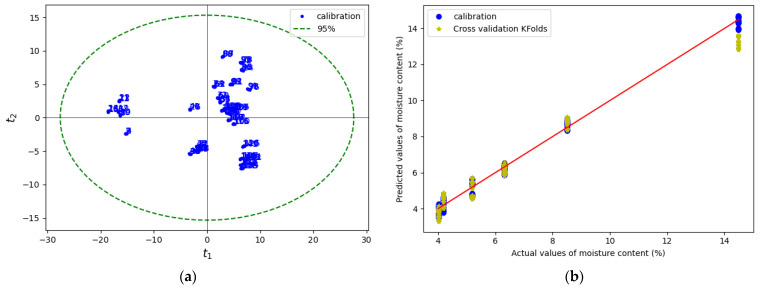
(**a**) PCA Hotelling score plot of pretreated data and (**b**) PLS regression model of actual and predicted values of tablet surface water content.

Water molecules that diffuse from the powder bed can be trapped at the interfaces between the compression tools and powder bed during compaction, which may cause water vapor to accumulate in these regions. To verify this assumption, an NIR sensor was used to measure the amount of moisture at the tablet surface just after ejection. Figure 18a shows the raw noisy NIR spectra collected for tablets with a water content of 4–14.5%. The raw spectra without pretreatment show a water absorption band in the ranges of 1190–1220 nm and 1400–1690 nm. After pretreatment, with the combination of EMSC and SG (17, 1, 0), weak and strong water absorption at 1202 nm and 1450 nm, respectively, was observed (Figure 20b). The PCA score plot coupled with the Hotelling T^2^ ellipse at a 95% confidence interval created with the pretreated data does not show outliers (Figure 21a). The model with three principal components explained 98% of the total variance of X (wavelength) and 98.5% of the total variance of Y (water content). A calibrated model (Figure 21b) was obtained with a regression coefficient in calibration (R^2^) of 97% and a root-mean-square error in calibration (RMSEC) of 0.14%. In the K-folds cross-validation, an R^2^ of 0.96% and RMSECV of 0.20% were obtained.

**Figure 22:**
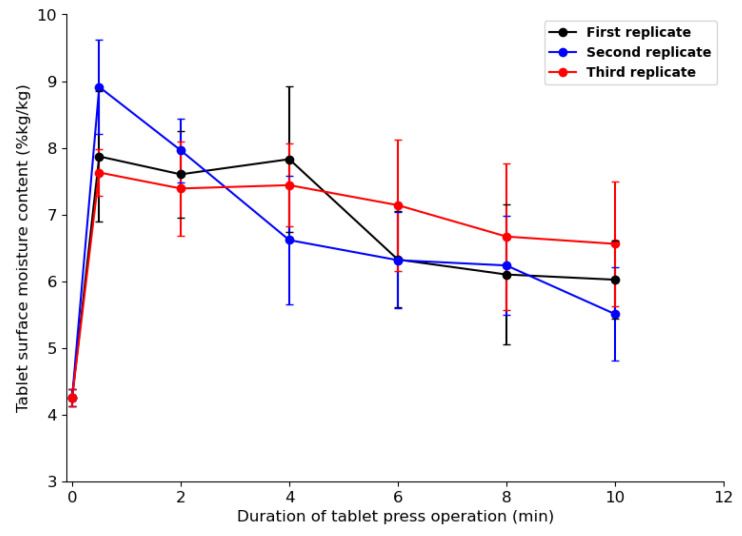
The predicted surface moisture content of ejected tablets during 10 min of tableting runs.

Figure 22 shows the predicted tablet surface moisture content for 10 min of tablet press operation, representing 600 tablets pressed. The tablets had the same compaction relative density of about 65%. All the tablets were compressed from the same batch of powder. Each point on Figure 22 represents the average moisture content of five tablets, with the measurements made every two minutes. Three replicate tests were performed, with two hours between each replicate to allow enough time for the tableting tools to cool down. ANOVA showed that the three replicates were not significantly different (*p* = 0.96). Figure 22 shows that at 0 min, the moisture level of the powder was the same as the ambient condition of 4.25%. After a half-minute run, the moisture content of the tablet surface increased from the ambient to 9% for the first replicate and 7.5% for the second and third replicates. The difference in moisture content between the replicates may be explained by the rate of moisture evaporation from the tablet surface after the ejection phase, which may vary due to differences in tablet surface temperature between replicates. The increase observed after a half minute of tableting may be attributed to the abrupt increase in tablet temperature (Figure 17), which led to the transfer of enough kinetic energy to water molecules in the tablet’s pore channels. Between the half minute and 10 min of tableting runs, the moisture content of the tablet surface decreased for the three replicates (Figure 22). The gradual rise in tablet temperature shown in Figure 17 may explain the observed decrease in moisture content, because as the tableting runs increases the evaporation from the tablet surface may become more important. However, the tablet surface temperature increased at a slower rate from the first tablet compression, which consequently led to a slower rate of decrease of the tablet surface moisture content. After 10 min of runs, a steady state was not reached because the tablet temperature was still rising.

Based on these observations, we may assume that the moisture evaporating from the tablet at the punch and tablet interface accumulates. The water molecules in the water vapor can be physiosorbed through van der Waals forces on the upper punch surface during the dwell time [84,85,86]. Due to the confined space between the powder bed and the upper punch, i.e., the interface, the number of van der Waals interactions between the vapor-phase molecules in that region can increase. Therefore, the first layer adsorbed on the punch surface may attract more water molecules, and multiple layers of water molecules may develop on the punch surface. The higher amount of water vapor evaporating from the tablet may result in water vapor condensation below water vapor saturation pressure, which is known as capillary condensation [87,88,89]. Capillary condensation may occur at the interface between the surface and the tablet during the dwell time. On the punch surface, capillary condensation can be more pronounced in cavities, such as the valleys in surface roughness, logos cavities, and eventual cracks [87,88,89].

Capillary condensation at the punch surface during tableting can lead to capillary adhesion of the tablet surface particles to the upper punch surface [37,90,91]. The tablet’s surface particles can adhere to the punch surface when the capillary forces between the punch surface and the particles are stronger than the cohesive forces between the tablet’s particles at the surface and the core [92]. Moisture is an important factor that contributes to the sticking of tablets observed during tableting runs [37,41,43]. Granules with higher moisture content led to a higher frequency of sticking [37]. Similarly, tableting parameters such as compression speed increase the occurrence of sticking [93,94]. In addition, compression speed increases the heat generated during tableting [18,23]. Consequently, the greater occurrence of sticking could be attributed to the increased water vapor at the punch–tablet interface due to the higher heat generated with increased speed. Furthermore, punches with surface cavities, such as logos and cracks, due to surface wear have been found to increase sticking [1,35,79], which may be attributed to the capillary condensation in these cavities, where capillary bridges between the punch and particles cause adherence through capillary forces. The use of punches coated with hydrophobic materials has been shown to minimize or diminish the frequency of sticking [1,29]. This may be due to hydrophobic surfaces preventing water molecules from adsorption, leading to the avoidance of capillary condensation.

## 5. Conclusions

Finite element modeling of the compaction phase of the tableting process was performed to investigate the density, temperature, and moisture distribution and evolution of tablets. The DPC and HAM models implemented in COMSOL Multiphysics^®^ 5.6 software were used. At the end of the compaction phase, (1) relative density tended to be uniform across the tablet, but was higher at the tablet edges, and (2) the temperature of the powder bed increased during compaction and was higher at the center of the tablet than on the surface, which was much cooler due to heat dissipation through the compression tools. These thermomechanical simulation results are in broad agreement with the experimental results of this study and corroborate findings reported in the literature. The simulation results showed that powder moisture evaporated from the powder bed to its surroundings during the compaction phase. The NIR light (wavelengths: 970 nm to 1700 nm) penetration depth was found to be less than 450 μm into the MCC tablet. Hence, the NIR sensor was used to measure the water content of the tablet surface just after ejection, and showed that moisture content was higher at the tablet surface than the loose powder.

These results suggest that a fraction of the water molecules evaporating from the powder bed at the powder and punch interfaces accumulates on the tablet surface. In addition, the evaporation rate increased as the tableting runs increased due to the gradual rise in tablet temperature. Based on these observations, we propose that moisture evaporation from the powder bed and accumulation at the punch–tablet interfaces can induce capillary condensation between the tablet and the punch during the dwell time, leading to sticking. The results of this study bring new insights into punch sticking and may contribute to the investigation of other tableting problems.

## Data Availability

Not applicable.

## Supplementary Materials

The following supporting information can be downloaded at https://www.mdpi.com/article/10.3390/pharmaceutics15061652/s1: S1: Video of tablet surface moisture content measurement; S2: Drucker Prager Cap model parameters; S3: Heat transfer coefficients calculation.
